# Sustainability of return to work in sick-listed employees with low-back pain. Two-year follow-up in a randomized clinical trial comparing multidisciplinary and brief intervention

**DOI:** 10.1186/1471-2474-13-156

**Published:** 2012-08-25

**Authors:** Chris Jensen, Ole Kudsk Jensen, Claus Vinther Nielsen

**Affiliations:** 1 Department of Clinical Social Medicine, Public Health and Quality Management, Central Denmark Region and Section of Clinical Social Med. and Rehabilitation, School of Public Health, University of Aarhus, Aarhus, Denmark; 2 The Spine Center, Department of Internal Medicine, Regional Hospital Silkeborg, Silkeborg, Denmark

**Keywords:** Return to work, Sick leave, Relapse, Low back, Multidisciplinary

## Abstract

**Background:**

Sick-listed employees with low back pain had similar return to work (RTW) rates at one-year follow-up in a randomized trial comparing two interventions, but the effects were modified by specific workplace related factors. The present study addressed the sustainability of the intervention effects by performing a two-year follow-up and by using different outcome measures.

**Methods:**

A total of 351 employees sick-listed for 3–16 weeks due to LBP were recruited from their general practitioners and were randomly allocated to a hospital-based brief or multidisciplinary intervention. Outcome measures were based on sick leave registered in a national database of social and health-related benefits. RTW rates, RTW status, sick leave weeks and sick leave relapse were studied.

**Results:**

During the two-year follow-up 80.0% and 77.3% had RTW for at least four weeks continuously, and the percentages with RTW at the 104^th^ week were 61.1% and 58.0% in the brief and multidisciplinary intervention groups, respectively. At the 104^th^ week 16.6% and 18.8% were on sick leave in the two groups, respectively, and about 12% were employed in modified jobs or participated in job training. The number of weeks on sick leave in the first year was significantly lower in the brief intervention group (median 14 weeks) than in the multidisciplinary intervention group (median 20 weeks), but during the second year the number of weeks on sick leave were not significantly different between intervention groups. Subgroups characterised by specific work related factors modified the effect of the intervention groups on RTW rates (p = 0.017). No difference in sick leave relapse was found between the intervention groups.

**Conclusion:**

The effects of the brief and multidisciplinary interventions at the two-year follow-up were in general similar to the effects at one-year follow-up.

**Trial Registration:**

Current Controlled Trials ISRCTN18609003

## Background

We have previously reported the results of a one-year follow-up of a randomized trial that compared a brief and a multidisciplinary intervention to facilitate return to work (RTW) and improve function in patients on sick-leave due to low-back pain (LBP) [1,2]. RTW, disability and pain status were not different in the two intervention groups at the one-year follow-up. However, a subgroup of patients who had some influence over their work and its planning and had no fear of losing their job due to sick leave returned more quickly to work if they received the brief intervention than if they received the multidisciplinary intervention [2]. The employees who had no influence over their work or feared losing their job appeared to return more quickly to work if they received the multidisciplinary intervention.

“RTW” was defined as the first period of four consecutive weeks without receiving health-related compensation benefits (primarily sick leave compensation). Four weeks without sickness relapse was considered a period sufficiently long to suggest stability and lasting work functioning. The definition was a pragmatic one and it is in line with that used in previous Dutch studies on RTW interventions [3-6]. Evaluation of the sustainability of RTW may, however, require a broader range of outcome measures and a longer follow-up period than that used in previous studies. Although outcomes in RTW research on sick-listed employees with musculoskeletal problems have differed, a measure of time on sick leave until RTW has been fairly consistently deployed. The first four-week period without sick leave has been used to indicate termination of the “time to RTW” in several studies, [3-6]. Another study has used a similar principle without defining the duration of the period without sick leave [7]. Others have used RTW status at a predefined time point after patient inclusion, typically one year. For instance, Haldorsen et al. [8] used benefit payment status once a month as an outcome measure, whereas Bültmann et al. [9] reported status at three, six and 12 months as secondary outcome measure. Hagen et al. [10] used a similar approach to analyze follow-up for up to three years. Total numbers of hours, days or weeks with sick leave have also been used, e.g. total number of hours in specific periods after an intervention [9], total number of days [3,10,11] or a similar measure such as total amount of sick leave benefits [12].

Second, sustainability of RTW requires stability over time. One of the first RTW studies on the long-term effects of brief clinical intervention with reassuring advice in LBP patients reported that the effects lasted for at least five years [13]. Thus, 19% in the intervention group and 34% in the primary care control group were still on sick leave after five years. Loisel et al. [12] reported that substantial cost-benefits were associated with the intervention known as the Sherbrooke model at six years of follow-up. This multidisciplinary intervention consisted of a combined workplace and clinical intervention. Hagen et al. [10] performed a study on a similar brief clinical intervention aiming to facilitate RTW as that of Indahl et al. [7] and replicated the intervention effects at the one year follow-up, but they found no difference between the intervention and the control group in primary care in the second and third year of follow-up. The number of days on sick leave for the entire three-year follow-up period was significantly lower in the intervention group than in the control group, even though the difference lay only in fewer absence days during the first year of follow-up.

Third, sustainability may be measured in terms of relapse as low back pain is often an episodic event. In the study of Hagen et al. 62% reported new episodes of sick leave due to low back pain during a three-year follow-up period [10]. Thus, interventions aiming to treat pain, to teach the patient to cope with pain or to modify the environment (e.g. work) may also affect the risk of sick leave relapse. Relapse may be studied in terms of the number of new episodes in the follow-up period or by reporting the total number of sick leave days during the follow-up period [6], which also is the basis for cost-benefit or cost-effectiveness analyses [8,9,12]. Some intervention studies have reported both outcome measures [5,10].

The aim of the present paper was to study the sustainability of RTW in a trial comparing a brief and a multidisciplinary intervention in patients with sick-leave due to LBP. The results at one and two years of follow-up were compared. Three different outcomes were used: 1) Duration until RTW during the first and second year, 2) number of weeks on sick leave and 3) RTW status recorded at the 52^nd^ and 104^th^ week of follow-up. Also, the effect of interventions on sickness relapse during the second year was analyzed in those who had returned to work within the first year after the intervention.

## Methods

### Study design and participants

This is a two-year follow-up of a randomized trial comparing a brief and a multidisciplinary intervention to facilitate RTW. The inclusion criteria were: 1) sick leave for 3–16 weeks due to LBP; 2) 16–60 years of age; and 3) ability to read and speak Danish. The exclusion criteria were: 1) unemployment; 2) continuing or progressive symptoms indicating plans for surgery; 3) surgery in the spine within the past 12 months; 4) diagnosis of specific back disease (e.g. tumour); 5) diagnosis of primary psychiatric disease; 6) pregnancy; or 7) known substance abuse. Patients from nine municipalities in Central Denmark Region were referred to The Spine Center by their general practitioner (GP). The inclusion and exclusion criteria were evaluated at The Spine Center, Region Hospital Silkeborg, where the study was performed. A total of 351 patients were enrolled in the study.

### Interventions

The interventions have previously been described [1]. In short, the sick-listed patients underwent a clinical examination by a rehabilitation doctor and a physiotherapist at the first visit at the clinic and were randomly allocated to either brief or multidisciplinary intervention based on block randomization. Magnetic resonance imaging of the lumbar spine was performed in 75% of patients and 10% in each intervention group had surgery after enrolment in the study, most often for disc herniation with radiculopathy.

Reassuring explanations for back and leg pain and advice to gradually increase physical activity were provided during the examination, which lasted for about two hours. After two weeks, all participants were scheduled for an informational follow-up visit with the physiotherapist and most were scheduled for a follow-up visit with the rehabilitation doctor. If allocated to the brief intervention, the participant continued treatment and rehabilitation with his or her general practitioner (GP). For participants allocated to the multidisciplinary intervention, a visit was scheduled with a case manager. The case manager conducted a comprehensive interview covering aspects of work and private life and designed a tailored rehabilitation plan to facilitate the employee’s RTW. The rehabilitation plan was discussed by the entire team at The Spine Center, which counted a specialist of social medicine, a specialist of rheumatology and rehabilitation, a physiotherapist, a social worker and an occupational therapist. The case manager also contacted the work place and the municipal job centre to discuss and coordinate relevant initiatives. In Denmark, all employees are entitled to sick leave compensation from the employer or the local municipality. After two weeks of sick leave (now four weeks), the job centre administration in the municipality paid compensation to the employer or directly to the sick-listed employee (regulated by labour market agreements). This compensation is financed by the tax-payers. The case manager was employed at the hospital and worked independently from the job centre. The main task of the case manager was to coordinate RTW initiatives based on knowledge of legislation, workplace conditions and the health status of the participants. The case manager could arrange meetings between the participant and each of the other specialists, meetings at the work place and meetings with the job centre, if relevant.

### Variables

A baseline questionnaire with questions on health issues, functioning as well as work-related and basic socio-demographic factors was completed by all patients.

Outcome measures were RTW and weeks on sick leave. RTW was measured in two ways: 1) Duration until the first four week period that the patient did not receive sick leave benefits (or other health-related social benefits); and 2) RTW at one year and two years which was defined as receiving no social or health-related benefits (except unemployment benefit) in the 52^nd^ and 104^th^ week after inclusion (irrespective of benefits received before these specific weeks). Other outcome categories were sick leave (partial or full), modified job or training and labour market exclusion (early retirement). Data on sick leave compensation were obtained from a national database administered by the Ministry of Employment. This database includes information on all public transfer payments (social and health-related benefits) for all Danish citizens registered on a weekly basis since 1991 [14]. Reasons for sick leave or other health data are not available in the database, but such information was obtained at the first interview with the participants in the clinic.

### Analyses

Outcome measures were analyzed for the intervention groups in the total sample of participants (n = 351) and for subgroups as described in a previous paper [2]. In short, the subgroups were identified by performing tests for interaction between intervention group and 18 different work-related factors in Cox regression analyses on RTW. The work-related factors were assessed in questionnaires at baseline and the analyses were adjusted for age, gender and other work-related factors. Three factors (job satisfaction, influence on work planning and risk of losing job due to sick leave) were identified which showed interaction with the intervention groups, i.e. the hazard ratios of RTW in the brief/multidisciplinary intervention groups were different for responders with low and high job satisfaction, low and high influence and low and high risk of losing job, especially when work injury claimants were excluded. Considerable overlap between these work-related variables were found and we eventually formed two subgroups based on a combination of several factors. The subgroups were verified in a new study group based on 120 new participants. Subgroup 1 comprised participants who reported that they had influence on the planning of their work and who did not feel at risk of losing their job because of their sick leave. Subgroup 2 comprised participants reporting no influence on work planning and/or reporting at risk of losing their job because of sick leave. Furthermore, work injury claimants (n = 83) were included in Subgroup 1 as they showed slower RTW in the multidisciplinary intervention group irrespective of their answers to the other questions.

In survival analyses RTW was defined as the first uninterrupted period of work during which no social or health-related benefits were received by the patient within the follow-up period except unemployment benefits. RTW was thus defined as the first four consecutive weeks without benefits to calculate hazard rate ratios (HRRs) and 95% confidence intervals (95% CI) adjusted for gender and age for multidisciplinary versus brief intervention using Cox regression models.

Furthermore, the percentages with RTW in the follow-up period were calculated for other durations than four weeks without benefits as definition of RTW.

The duration of sick leave was calculated by counting the total number of weeks with sick leave during the first and second years of follow-up. Differences between intervention groups were not normally distributed and they were tested with the Wilcoxon rank-sum test.

The percentages with RTW recorded at the 52^nd^ week (one-year follow-up) and 104^th^ week (two-year follow-up) after their inclusion were also calculated. The percentages with sick leave, in modified jobs, excluded from the labour market or with other social income benefits were also calculated. Logistic regression analyses were used to test for differences in RTW status between interventions adjusted for gender and age.

For those who returned to work within the first year, the number of weeks with recurrent sick leave during the second year was calculated. Differences between intervention groups were tested with the Wilcoxon rank-sum test.

A p-value of 0.05 was considered statistically significant. The software package STATA 11.1 was used for statistical analyses.

### Ethical approval

The study was discussed with the regional research ethics committee. Approval was not considered necessary by the committee because all participants received the best available clinical care and no biological material was involved. All participants signed informed consent. The study was approved by the Danish Data Protection Agency (No. 2007-41-1278).

## Results

A total of 351 patients were randomized to brief (n = 175) or multidisciplinary intervention (n = 176). In the brief intervention group the mean age of the patients was 41.9 (SD = 10.4) years and 50.3% were women. In the multidisciplinary intervention group the mean age of the patients was 42.1 (SD = 10.5) years and 54.0% were women. Seven patients dropped out after randomization, but they were included in the analyses as outcome data were available for all patients. However, another seven patients had not answered the questions which were used to form the subgroups at baseline. Therefore, the numbers of patients were 227 and 117 in Subgroup 1 and 2, respectively.

The fractions of patients with RTW for at least four weeks during the two-year follow-up period were not statistically significantly different in the brief and multidisciplinary intervention groups (Table 1). The estimated number of subjects who returned to work decreased linearly with an increase in the required duration of the period without sick leave. However, the relative chance of RTW associated with the two interventions was similar for different durations, and the chances of RTW were not statistically significantly different between the intervention groups (Table 1). When a four-week duration without sick leave compensation was used to define RTW, 76% and 72% accomplished RTW during the first year in the brief and multidisciplinary intervention groups, respectively (Table 2).

**Table 1:** Impact of the duration of the period without benefits after sick leave on number of patients with return to work (RTW) in the first year based on survival analyses

**Number of consecutive weeks without sick leave**	**Participants with RTW**		**HRR (Multidisciplinary/brief intervention)**
1	267	76.1%	0.86 (95% CI: 0.68-1.10)
4	260	74.1%	0.85 (95% CI: 0.67-1.09)
12	242	69.0%	0.82 (95% CI: 0.63-1.05)
26	204	58.1%	0.82 (95% CI: 0.62-1.08)

**Table 2:** Return to work (RTW) and sick leave outcomes in the brief (N = 175) and multidisciplinary intervention groups (N = 176)

	**One-year follow-up**			**Two-year follow-up**		
	**Brief intervention**	**Multidiscipl. intervention**	**P**	**Brief intervention**	**Multidiscipl. intervention**	**P**
**RTW >4 weeks** during follow-up (n;%)	133; 76.0	127; 72.2	0.20*	140; 80.0	136; 77;3	0.22*
**Status at 52**^**nd**^**and 104**^**th**^**week**						
RTW (n;%)	115; 65.7	108; 61.4	0.43**	107; 61.1	102; 58.0	0.54**
Sick leave (n;%)	44; 25.1	49; 27.8		29; 16.6	33; 18.8	
Modified job or training (n;%)	10; 5.7	16; 9.1		21; 12.0	22; 12.5	
Labour market exclusion (n;%)	6; 3.4	1; 0.6		11; 6.3	10; 5.7	
Other (n;%)	0; 0.0	1; 0.6		6; 3.4	7; 4.0	
Died or moved abroad (n;%)	0; 0.0	1; 0.6		1; 0.6	2; 1.1	
**Sick leave weeks in first and second year** (25; 50; 75 percentiles)	5; 14; 37	10; 20; 43	0.018***	0; 0; 14	0; 1; 17	0.29***

*Cox regression model adjusted for gender and age.

**Logistic regression model adjusted for gender and age.

***Wilcoxon rank-sum test.

The number of weeks on sick leave in the first year was statistically significantly lower in the brief intervention group (median 14 weeks) than in the multidisciplinary intervention group (median 20 weeks, Table 2).

The RTW status registered in the 52^nd^ week after the first visit were not statistically significantly different in the in the brief and multidisciplinary intervention group, 66% and 61% respectively. (Table 2). Among those who were not healthy enough to return to normal work, most were on sick leave, but some had modified work and a few had become excluded from the labour market and were receiving early retirement or temporary social benefits (Table 2). Sick leave status was observed for 93 participants in the 52^nd^ week and 71% of these patients had uninterrupted sick leave since the first visit at the Spine Center, i.e. sick leave after 52 weeks was known to be due to LBP. This was only the case for 32% of 62 patients on sick leave at the 104^th^ week.

The subgroup analyses showed a better effect for all three outcome measures in “Subgroup 1” by brief intervention as compared to the multidisciplinary intervention, but RTW status in the 52^nd^ week was not statistically significantly different between the intervention groups (Table 3). In “Subgroup 2”, the tendency was in the opposite direction, even if the differences fell short of reaching the level of statistical significance (Table 4). “Survival curves” based on analyses over a two-year period showed that a few more patients had succeeded in RTW than during the first year (Table 2) (HR 0.86; 95% CI: 0.68-1.09).

**Table 3:** Return to work (RTW) and sick leave outcomes in the brief and multidisciplinary intervention groups within Subgroup 1 (those with influence on the planning of their own work and no perceived risk of losing job and/or being a work injury claimant)

	**One-year follow-up**			**Two-year follow-up**		
	**Brief intervention N = 113**	**Multidiscipl. intervention N = 114**	**P**	**Brief intervention N = 113**	**Multidiscipl. Intervention N = 114**	**P**
**RTW >4 weeks** during follow-up (n;%)	90; 79.7	78; 68.4	0.014*	92; 81.4	85; 74.6	0.028*
**Status at 52**^**nd**^**and 104**^**th**^**week**						
RTW (n;%)	77; 68.1	66; 57.9	0.12**	74; 65.5	62; 54.4	0.068**
Sick leave (n;%)	28; 24.8	39; 34.2		14; 12.4	27; 63.7	
Modified job or training (n;%)	6; 5.3	8; 7.0		15; 13.3	14; 12.3	
Labour market exclusion (n;%)	2; 1.8	0; 0.0		6; 5.3	6; 5.3	
Other (n;%)	0; 0.0	0; 0.0		3; 2.7	3; 2.6	
Died or moved abroad (n;%)	0; 0.0	1; 0.9		1; 0.9	2; 1.8	
**Sick leave weeks in first and second year** (25; 50; 75 percentiles)	6; 14; 34	12; 26; 51	0.001***	0; 0; 14	0; 1; 25	0.11***

*Cox regression model adjusted for gender and age.

**Logistic regression model adjusted for gender and age.

***Wilcoxon rank-sum test.

**Table 4:** Return to work (RTW) and sick leave outcomes in the brief and multidisciplinary intervention groups within Subgroup 2 (those without influence on the planning of their own work or feeling at risk of losing job and not a work injury claimant)

	**One-year follow-up**			**Two-year follow-up**		
	**Brief intervention N = 57**	**Multidiscipl. intervention N = 60**	**P**	**Brief intervention N = 57**	**Multidiscipl. Intervention N = 60**	**P**
**RTW >4 weeks** during follow-up (n;%)	38; 66.7	48; 80.0	0.09*	43; 75.4	50; 83.3	0.17*
**Status at 52**^**nd**^**and 104**^**th**^**week**						
RTW (n;%)	33; 57.9	41; 68.3	0.19**	29; 50.9	39; 65.0	0.098**
Sick leave (n;%)	16; 28.1	10; 16.7		14; 24.6	6; 10.0	
Modified job or training (n;%)	4; 7.0	8; 13.3		6; 10.5	8; 13.3	
Labour market exclusion (n;%)	4; 7.0	0; 0.0		5; 8.8	3; 5.0	
Other (n;%)	0; 0.0	1; 1.7		3; 5.3	4; 6.7	
Died or moved abroad (n;%)	0; 0.0	0; 0.0		0; 0.0	0; 0.0	
**Sick leave weeks in first and second year** (25; 50; 75 percentiles)	6; 13; 40	6; 14; 30	0.59***	0; 2; 15	0; 2; 15	0.62***

*Cox regression model adjusted for gender and age.

**Logistic regression model adjusted for gender and age.

***Wilcoxon rank-sum test.

The RTW status registered in the 104^th^ week after the first visit showed RTW percentages that were lower than those registered in the 52^nd^ week; but like in the 52^nd^, the differences between the two intervention groups were not statistically significantly different (Table 2). In both intervention groups, more patients had modified jobs or were excluded from the labour market at the two-year follow-up than at the one-year follow-up.

The number of weeks on sick leave was lower during the second year than during the first year and it did not statistically significantly differ between the two intervention groups (Table 2). The survival analyses at the two-year follow-up showed a better effect in “Subgroup 1” by brief intervention as compared to the multidisciplinary intervention, but RTW status in the 104^th^ week was not statistically significantly different (Table 3). In “Subgroup 2”, the differences in RTW between interventions at the two-year follow-up were in the opposite direction to those of “Subgroup 1”, but they were not statistically significant (Table 4).

Effect modification analyses based on Cox regression adjusted for gender and age where the patients were categorized with respect to “Subgroup 1” and “Subgroup 2” showed a statistically significant modification on the effect of brief and multidisciplinary intervention both at the one-year (p = 0.006) and the two-year follow-up (p = 0.017). In these analyses, RTW was defined as four consecutive weeks without benefits, and at both follow-up times, the brief intervention appeared more effective than the multidisciplinary intervention in “Subgroup 1”, and the opposite pattern was present in “Subgroup 2”.

For those who experienced RTW during the first year, recurrent sick leave was monitored during the second year of follow-up. New episodes of sick leave were experienced by 42% of the participants. Table 5 shows that the median number of sick leave weeks was 0 in the second year in both intervention groups, but 25% (75 percentile) experienced at least 7 and 11 weeks of sick leave in the brief and multidisciplinary intervention groups, respectively. The difference in the number of new episodes between the intervention groups was not statistically significant, and similar patterns were found in the subgroups. The mean numbers of new weeks with sick leave were 6.5 and 7.8 in the brief and the multidisciplinary intervention group, respectively.

**Table 5:** Recurrence of sick leave in the second year after the start of the intervention for subjects who accomplished return to work during the first year (N = 260)

		**Brief intervention**		**Multidiscipl. intervention**		
		**25; 50; 75 percentiles**	**n**	**25; 50; 75 percentiles**	**n**	p*
**All participants**	Sick leave weeks	0; 0; 7	133	0; 0; 11	127	0.42
**Subgroup 1**^#^	Sick leave weeks	0; 0; 7	90	0; 0; 12	78	0.49
**Subgroup 2**^##^	Sick leave weeks	0; 0; 7	38	0; 0; 9	48	0.84

^#^Subgroup 1: Influence on planning own work, no perceived risk of losing job and/or work injury claimant.

^##^Subgroup 2: No influence on planning own work or feeling at risk of losing job.

*Wilcoxon rank-sum test.

## Discussion

### Different outcome measures

The results of the present randomized comparative trial depended partly on the outcome measure chosen. At the one-year follow-up, the number of weeks on sick leave was statistically lower in the brief intervention group than in the multidisciplinary group which indicated that this intervention was the more effective. The other two outcome measures showed the same tendency, but the differences were not statistically significantly different. Even if the conclusion thus depends on the outcome measure chosen it remains clear that all three outcome measures pointed in the same direction.

At the two-year follow-up, the relative effects of the two interventions were similar to those obtained at the one-year follow-up. The “survival analyses” showed that about 5% more patients had achieved a four week RTW period during the second year, but this was the case for both intervention groups. The number of sick leave weeks was much lower in the second year than in the first year; again, this was the case for both intervention groups. The percentages of former patients having resumed work were not statistically different between the intervention groups at any of the follow-up points. However, it should be noted that the percentage at work in the 104^th^ week was slightly lower (approximately 60%) than the percentage at work in the 52^nd^ week (approximately 64%). The lower prevalence of sick leave in the second year thus testifies to the sustainability of RTW, but the slightly lower fraction of employees with regular work in the second year may be ascribed to the fact that more employees were engaged in modified work or were no longer part of the labour market due to early retirement or for other reasons. The percentage of patients with RTW at the two-year follow-up was higher than the percentage at the one-year follow-up because patients with RTW could not enter the study again if they had new sick leave spells. The number of RTW events will therefore either remain constant if no new patients return to work or increase if at least one new RTW event is registered. It is therefore crucial to any comparison of RTW rates between studies that the outcome measures are exactly the same.

Our “survival curves” showed that 74% returned to work during the first year, which may be compared with the results of Dutch studies that also used a four-week period without sick leave in their definition of RTW. Anema et al. [6] reported that 91% of their intervention group accomplished RTW during the one-year follow-up period, and Heymans et al. [5] reported that approximately 80% of their intervention groups achieved RTW during their six-month follow-up period. The RTW rates in our study were also lower than those in the control groups of the two Dutch studies. This indicates that the Dutch context may facilitate RTW better than the Danish context. A more elaborate dismissal protection legislation in Holland than in Denmark may lay at the root of this difference. However, “usual care” in Holland is also different from that in Denmark, as Holland operates a system where an occupational practitioner deals with sick leave problems, whereas in Denmark, the general practitioner is the primary health professional involved. Furthermore, one third of our patients had radiculopathy and 10% in each intervention group had surgery, most often due to radiculopathy [1], whereas the Dutch study only included patients with non-specific LBP. A comparison with Norwegian studies is also difficult as outcome measures were defined differently [10,15].

It is possible that our interventions were less effective than usual care in Denmark. However, we consider this unlikely as an early intervention including a thorough clinical examination and reassuring advice is considered beneficial and has proven effective in other countries [6;10;12]. The only Danish study that could be used for comparison reported that at the 12-month follow-up, 78% were at work and 22% were on sick leave in the intervention group, and 62% were at work and 38% on sick leave in the primary care control group [9]. These figures may be compared with our status at the one-year follow-up, where 25% and 28% were on sick leave in our two intervention groups. Our percentages at work in the 52^nd^ week, i.e. 66% and 61%, were lower than the percentage of 78% reported in the previous Danish study. However the definition of RTW may have differed between our study and the previous Danish study; moreover, we separately measured other possible outcomes, like for instance “modified job or training”, which we do not know if was the case in the previous Danish study.

### Subgroups

A major weakness of conclusions based on a comparison of the brief and multidisciplinary intervention groups was the existence of subgroups in which the interventions seemed to affect return to work rates in opposite directions. The stratified analyses made clear that the brief intervention was statistically significantly more effective than the multidisciplinary intervention in “Subgroup 1” in which the patients reported to have influence on work planning and were not at risk of being dismissed. We previously reported that effect modification was present at the one-year follow-up; that is, the multidisciplinary intervention was more effective than the brief intervention in the other subgroup without job control or where the patients felt at risk of losing their job. The differences between the intervention groups were not statistically significantly different if analysis was confined to “Subgroup 2”, even if the average differences were similar to those found in the other subgroup. The reason for this lay in the difference in statistical power due to the lower number of patients in “Subgroup 2”. The higher number of patients in “Subgroup 1” was also the most important reason for the tendency towards a better effect of the brief intervention in the total sample of subjects, i.e. the brief intervention was more effective in about two-thirds of the patients, which pushed the average result in favour of the brief intervention. For the other one-third of the patients, the relative benefits of the multidisciplinary intervention seemed just as large. The correct conclusion of the study would probably be that the brief intervention worked better for about two-thirds of the patients, and the multidisciplinary intervention was more effective for the remaining one-third of the patients. This result was based on post-hoc subgroup analyses in the randomized trial, and it therefore should be verified in a randomized trial stratifying patients into appropriate subgroups before randomization.

Heymans et al. [5] reported higher RTW rates and less sick leave days in a group receiving low-intensity back-school intervention than in a group receiving high-intensity back-school intervention or usual care. High-intensity back-schools were not superior to usual care. However, only some of the analyses showed significant differences between groups, and subgroup analyses were not performed. Others have compared low-intensity interventions with usual care [7,10] or more intensive interventions with usual care [6,9,16], and positive effects have been reported for both types of interventions. In a recent review of randomized controlled trials, it was suggested that brief interventions (<12 hours spent on the intervention) were more effective than interventions where the efforts were more extensive, at least as compared with efforts lasting more than 32 hours [17]. Our subgroup analyses and those of others [8,18] indicated that it is very likely that both types of interventions may be effective, but that the effectiveness depends on other risk factors than those that were used to include sick-listed employees in the RCTs. The identification of such factors is important to the provision of the right kind of “treatment”. However, these factors may differ between different countries, occupational groups and so forth. The tendency for the brief interventions to be more effective than more intensive interventions may be explained in two ways. Like in our study, the subgroup who benefited more from the intensive intervention counted fewer members than the other subgroup, i.e. those who lacked job control and who were at risk of losing their jobs, which was reported by one-third. In Dutch studies, the risk of being dismissed during sick leave was probably much lower than in our study as the labour market legislations differ between Denmark and Holland. Like in Holland, dismissal protection is high in Norway, but low job control and other adverse factors that may require more intensive RTW efforts may be equally prevalent in all three countries. The other explanation lies in the duration of the intervention. The duration of a multidisciplinary intervention is longer than the duration of a brief one and may postpone RTW even if patients are, indeed, told to RTW as soon as possible during the course of the intervention.

We ascertained the same effect of intervention, whether brief or multidisciplinary, in the one-year follow-up and the two-year follow-up. In both subgroups, the outcome that measured work status at the 104^th^ week featured the largest differences between the interventions, as the percentages of patients at work were significantly different between intervention groups in”Subgroup 1”. In”Subgroup 2”, the same tendencies were seen at the two-year and the one-year follow-up and the percentage with sick leave in the 104^th^ week was considerably lower in the multidisciplinary intervention group than in the brief intervention group. Thus, the reported subgroup differences appeared sustainable.

### Sickness relapse

Long-term sickness relapse was not common within the first two years after the intervention was initiated. More than half of the employees who accomplished RTW did not experience new episodes of long-term sick leave, neither in “Subgroup 1” nor in “Subgroup 2” irrespective of intervention. However, it should be noted that we only measured new spells with durations of more than two weeks’ absence. Shorter sick leave spells may be more common and previous reports of frequent sick leave relapse after RTW cannot be contradicted [10].

### Methodological considerations

The duration of the period without sick leave used to define RTW was crucial when estimating the percentage of patients returning to work during the follow-up period based on “survival analyses”. When choosing a longer duration than one week (or one day for that matter), such as four weeks which has been the “tradition” in Dutch studies, one is more certain that RTW is sustainable, i.e. that the employee is capable of staying at work without new sick leave spells. However, the percentage of patients with RTW decreased gradually when the required duration of the period without sick leave was increased from one week up till 26 weeks as indicated in Table 1. No threshold was observed that could have served to define the temporal boundaries of the concept of sustainability. More importantly, the relative effects of the two interventions on the RTW rates did not change when the duration of the period with sick leave was changed. Thus, to compare effects of RCTs, the duration of the period required for an individual to have fully returned to work to define RTW is probably of minor importance.

The most important strength of the present study was that the outcome measures were based on registers that ensured follow-up for all participants. The DREAM register was established by the Ministry of Employment to be able to monitor all social transfer income, that is, tax-paid benefits due to social or health-related events with economic consequences such as sick leave. This also explains why we could not measure sick leave spells shorter than two weeks as this period is paid by the employer or the employee without compensation from the tax-paid public insurance system. Another shortcoming of the DREAM register is that the cause of sick leave is not registered. Thus, the cause was registered at the first visit at the Spine Center only for the initial sick leave which was required for being enrolled in the study. The cause of sick leave relapse was not known.

## Conclusions

The effects of the brief and multidisciplinary interventions at the two-year follow-up were similar to the effects reported at the one-year follow-up. A lower number of sick leave weeks was found in the brief intervention than in the multidisciplinary intervention group and the other outcome measures pointed in the same direction even though RTW rates were not statistically significantly faster in the brief intervention group. Long-term sickness relapse after RTW was not frequently observed in any of the intervention groups.

## Competing interests

The authors have no competing interests.

## Authors' contributions

CJ conceived the study, carried out the statistical analyses and drafted the manuscript. OKJ was involved in providing the interventions. All authors participated in the design of the study, helped draft the manuscript, helped improve the analyses and interpret the results. All authors have approved the final manuscript.

## Pre-publication history

The pre-publication history for this paper can be accessed here:

http://www.biomedcentral.com/1471-2474/13/156/prepub
